# Modeling of mitral chordae’s length in echocardiography as a function of their manual measurement in the operating room

**DOI:** 10.1186/s13019-022-01816-8

**Published:** 2022-04-04

**Authors:** Mesut Gun, Misbaou Barry, Yohann Bohbot, Christophe Tribouilloy, Gilles Touati

**Affiliations:** 1 Department of Cardiology, Amiens Picardie University Hospital Center, 1 Rue du Professeur Christian CABROL, 80054 Amiens Cedex 1, France; 2 Department of Cardiac Surgery, Amiens Picardie University Hospital Center, 1 Rue du Professeur Christian CABROL, 80054 Amiens Cedex 1, France

**Keywords:** Echocardiography measurement, Manual measurement, Correlation, Linear analysis, Prediction, Mitral tendinous chordae, Mitral valve repair

## Abstract

**Objective:**

In mitral insufficiency, trans-esophageal echocardiography (TEE) analysis of the mitral valve is an indispensable and irreplaceable examination to establish precisely the type of surgical repair to be performed and the exact length of neo-chordae to be used for an anatomical repair. The aim of our study is to find a predictive model of the Echographic Measurement (EM) variable according to the Manual Measurement (MM) variable of the mitral valve chordae, when the echocardiography measurement is not feasible.

**Patients and methods:**

This is a retrospective study on 191 patients undergoing mitral valve repair. The sex ratio (M/F) is 2.13 (130 men and 61 women). The collection of data of mitral chordae measurements performed echographically in preoperatively conditions, and then manually in intraoperatively conditions from January 2008 to December 2016 was made from the medical records of patients at the cardiology and cardiac surgery department of the University Hospital Center of Amiens in Picardy.

**Results:**

For this study 191 patients of mean age of 68 ± 13 years were included. The averages of the MM and EM of the mitral chordae were respectively 23 ± 2.5 mm and 24 ± 2.4 mm. The Pearson correlation coefficient was 0.897 (*p*-value < 10^−4^) showing a strong positive correlation between MM and EM. The results of the linear regression allow us to found the following mathematical model: *EM* = 0.87 × *MM* + 4.

**Conclusions:**

When patients have a contraindication to transesophageal echocardiography or when TEE is not feasible, manual measurement is performed during the surgery. By using the values obtained (MM) in the model, it is possible to predict the corresponding echographic measurements. This allows us to achieve the mitral tendinous chordae substitution with a very high precision.

*Trial registration*: Retrospectively registered.

## Introduction

The implantation of neo-chordae, consisting of replacing broken or elongated chordae with artificial chordae made of four-needle tresses of Gore-Tex®, is a recent technique for repairing the mitral valve [1] allowing, without any valve resection, to effectively correct mitral leakage while preserving the mobility of the two anterior and posterior valve leaflets. The length of these braids necessary to optimally correct mitral prolapse can be measured: either manually with the heart stopped during the extra-corporeal circulation (ECC), hypothermic and bloodless, without any pre- and post-load conditions, or by trans-esophageal echocardiography (TEE) performed in immediate preoperative which allows to predict, in a more physiological way, the length of these neo-chordae [2]. The echocardiographic measurement, made in systole during valve closure, corresponds to the distance between the top of the papillary muscle and the free edge of the non-prolapsed valve, opposite the valve leaflet, where the chordae is broken or elongated. This precise and reliable method of echocardiographic measurement should be preferred because it is carried out under physiological conditions of myocardial contractility. Some patients, however, have a contraindication to TEE:

- esophageal pathologies (stenosis, diverticulum, tumor, varicose veins, scleroderma),
- unstable lesions of the cervical spine,
- history of chest radiotherapy,
- massive hematemesis, progressive upper gastrointestinal haemorrhagic lesion, gastric or esophageal perforation,
- recent esogastric surgery,
- situations leading to the evaluation of a benefit / risk (shock, severe hypoxia),
- unfavorable anatomical conditions (poorly filled cardiac cavities, abnormal rotation of the atrioventricular floor, difficulty in exposing the papillary muscles) [3],
- anatomical variation in the number and shape of the pillars [4, 5]

All these contraindications require the performance of a manual intraoperative measurement [2]. The objective of this work was to find a model to predict echocardiographic measurement (EM) from manual measurement (MM).

## Patients and methods

### Study population

It is an observational, retrospective and monocentric study aiming to identify a model allowing to predict the EM from the MM in a population of patients having benefited from a surgical mitral valve repair by implantation of neo-chordae at the University Hospital Center of Amiens from Picardy.

The cordage measurement was performed manually and echocardiographically in 191 patients with severe mitral insufficiency and who had undergone mitral reconstruction surgery.

The study protocol was approved by the ethics committee of the University Hospital Center of Amiens in Picardie in France on 29 January 2013.

### Parameters studied

We measured these chordae first by trans-esophageal echocardiography (TEE), under general anesthesia as an immediate preoperative, then by manual intraoperative measurement with a graduated rule (heart stopped by cardioplegia, left atrium open). Myocardial protection was ensured by hyperpotassic and hypothermic cardioplegia (4 °C). The distance measured corresponds to the distance between the top of the papillary muscle and the free edge of the non-prolapsed valve opposite to the prolapse (ex. for segment P2 prolapse, measurement of the distance between the tip of the posteromedial pillar and the free edge of segment A2).

All the pre- and intraoperative data (MM, EM, age and sex) from the month of January 2008 to the month of December 2016 were extracted from the medical files of the patients of the medical and surgical cardiology department.

### Statistical analysis

We analyzed the data using SAS statistical version 7.1. In the descriptive study, we calculated absolute frequencies and relative frequencies (percentages) for the qualitative variables. We calculated means and standard deviations for the quantitative variables and analyzed the data to study the skewness of the distributions and outliers. We analyzed the normality of the two distributions by the tests of Shapiro–Wilk, Kolmogorov–Smirnov, Cramer-Von Mises and Anderson–Darling as well as their spread around the central value by checking the asymmetry coefficient (Skewness) and the coefficient flattening (Kurtosis) and another graphical appreciation of the fit, the quantile–quantile diagram, for the two distributions: manual measurement (MM) and echocardiographic measurement (EM). In the analytical study, the links between two quantitative variables were studied by the Pearson correlation coefficient. The univariate analysis was performed using simple linear regression to determine the mathematical model of the regression line.

In all the statistical tests, the significance level was fixed at a probability α: 0.05.

## Results

### General characteristics of the population

In total, we studied 191 patients with a male predominance (68%). The sex ratio (M/F) was 2.13 (130 men and 61 women). The mean age was 68 ± 13 years (21–91 years). Table 1 summarizes the characteristics of the population studied.

**Table 1:** Characteristics of study population (N = 191)

Characteristics	Values
Age—mean ± SD (years)	68 ± 13
Sex-ratio (M/F)	2.13
MM—mean ± SD (mm)	23 ± 2.50
EM—mean (mm)	24 ± 2.40
MM—minimum (mm)	16
MM—maximum (mm)	31
EM—minimum (mm)	17
EM—maximum (mm)	31.5

MM, Manual measurement; EM, echographic measurement; mm, milimeter

**Fig. 1:**
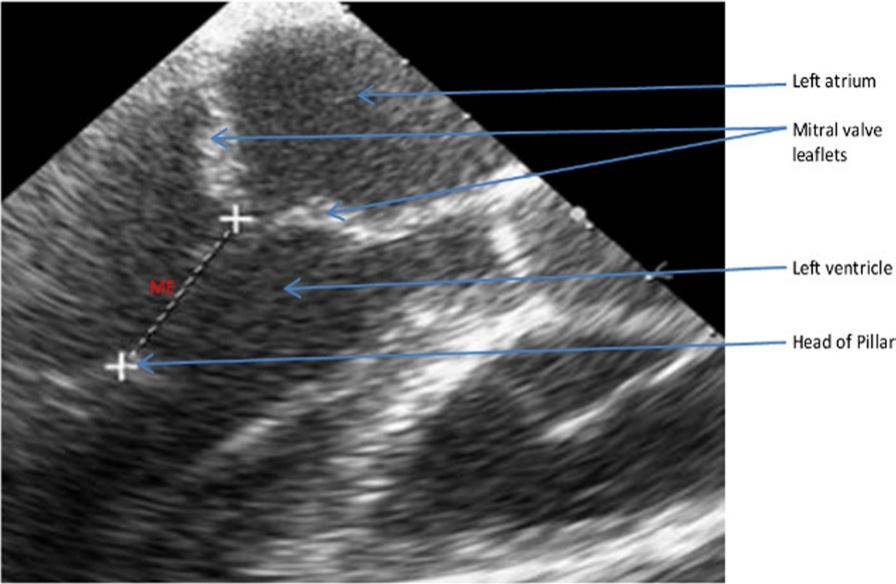
Echographic measurement (ME) of the mitral chordae

**Fig. 2:**
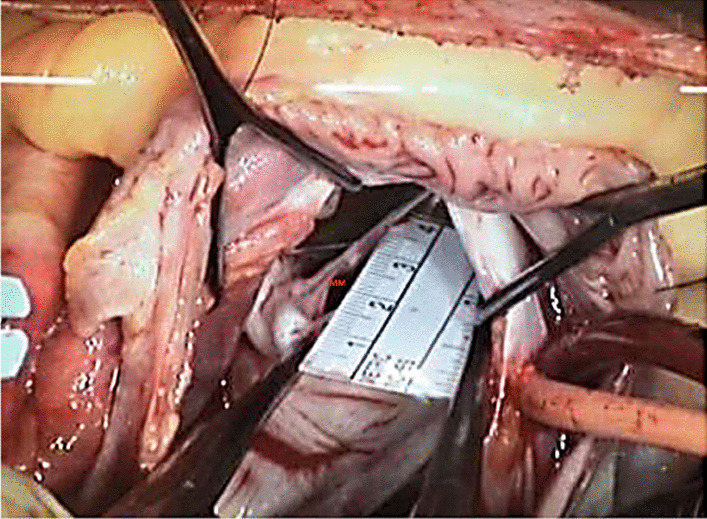
Manual measurement (MM) of mitral chordae

The means of preoperative echocardiographic (EM) measurements (Fig. 1) and manual intraoperative (MM) measurements (Fig. 2) of the mitral chordae were 23 ± 2.5 mm and 24 ± 2.4 mm respectively.

### Normality for manual measurement and echocardiographic measurement

We calculated the parameters (mean, standard deviation) of the variable MM and the variable EM (Table 1).

The normality of the quantitative variables MM and EM was verified by the tests of Shapiro–Wilk, Kolmogorov–Smirnov, Cramer-Von Mises and Anderson–Darling. The quantitative results which are detailed in Table 2 revealed perfect normality (*p*-value > 0.05) for each of the tests which therefore allowed acceptance of the null hypothesis H0 and rejection of the alternative hypothesis H1.

**Table 2:** Normality tets of manual measurements and echographic measurements

Test	*P*-value—MM	*P*-value—EM
Shapiro–Wilk	0.11	0.81
Kolmogorov–Smirnov	0.08	0.15
Cramer–Von Mises	0.14	0.25
Anderson–Darling	0.11	0.25

**Fig. 3:**
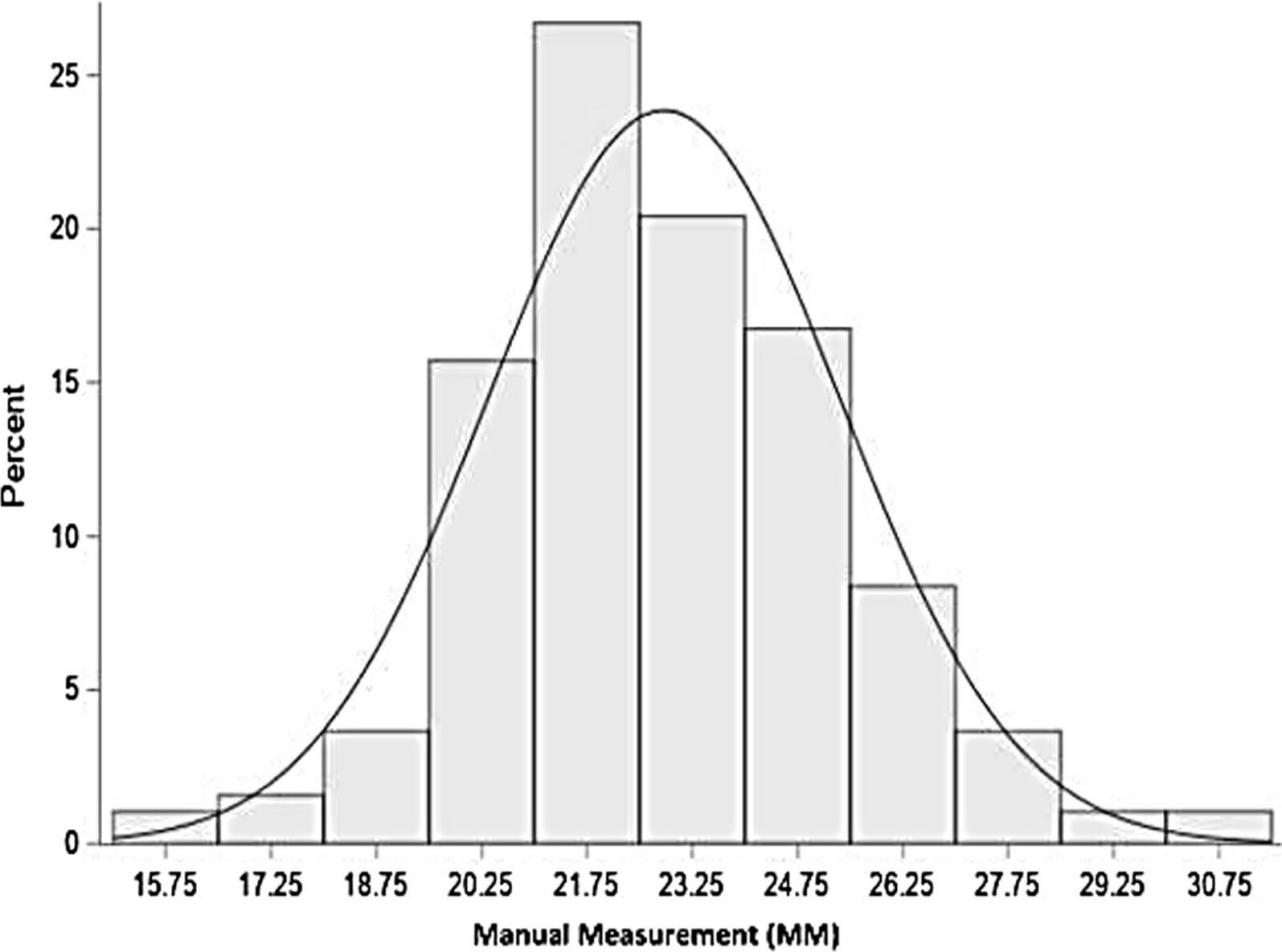
Distribution of manual measurements (millimeter)

**Fig. 4:**
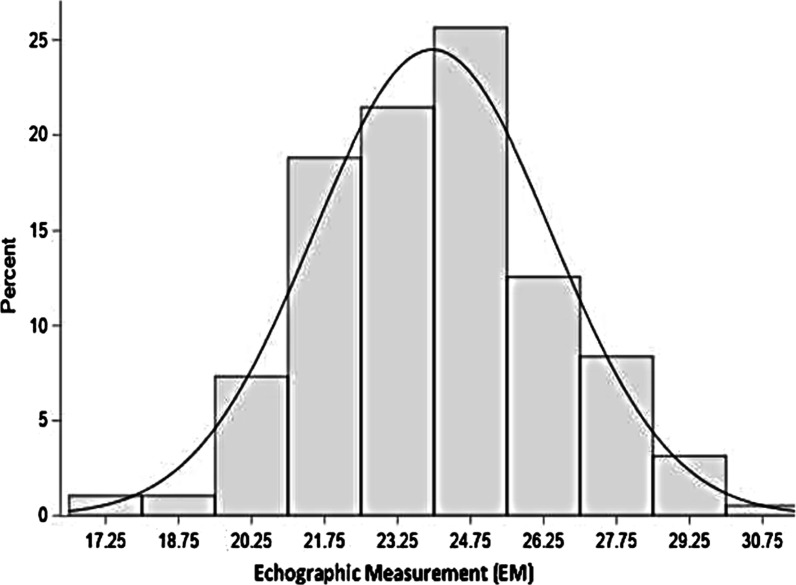
Distribution of echographic measurements (millimeter)

Their spread around the central value was checked by the asymmetry coefficient (Skewness → SMM: 0.33; SME: 0.08) and the flattening coefficient (Kurtosis → KMM: 0.61; KME: 0, 17) (Figs. 3 and 4).

### Correlation between echocardiographic measurement and manual measurement

We then verified and studied the statistical significance by a correlation with the Pearson correlation coefficient (r). For this, we posed the hypothesis H0 which signifies an independence (the inexistence of relation, H0: |r|= 0) against the alternative hypothesis H1 signifying a dependence between the two variables echographic measurement and manual measurement of the mitral cords (H1: |r|≠ 0), we set a probability of 0.05 as risk of error for the rejection of H0.

We looked for the absolute value of the Pearson correlation coefficient r (MM, EM) in the corresponding table of the correlation coefficient which corresponds to 0.1946 for N > 100 and α: 0.05. (See the table of correlation coefficient in the Appendix).

We calculated the theoretical value of the correlation coefficient r which is 0.897. We thus rejected H0 and retained H1 because r-critical (0.1946) is less than r-calculated (0.897 with *p* < 10^−4^) (Table 3).

**Table 3:** Pearson correlation statistics (r)

Variable	With variable	N	Sampling correlation	Confidence interval (95%)		*P*-value
EM	MM	191	0.897	0.865	0.921	0.0000

We have shown that the correlation coefficient is statistically significant because the calculated r is much higher than the critical r with a risk α: 0.05 and with degrees of freedom N-2 (191 inclusions-2: 189).

**Fig. 5:**
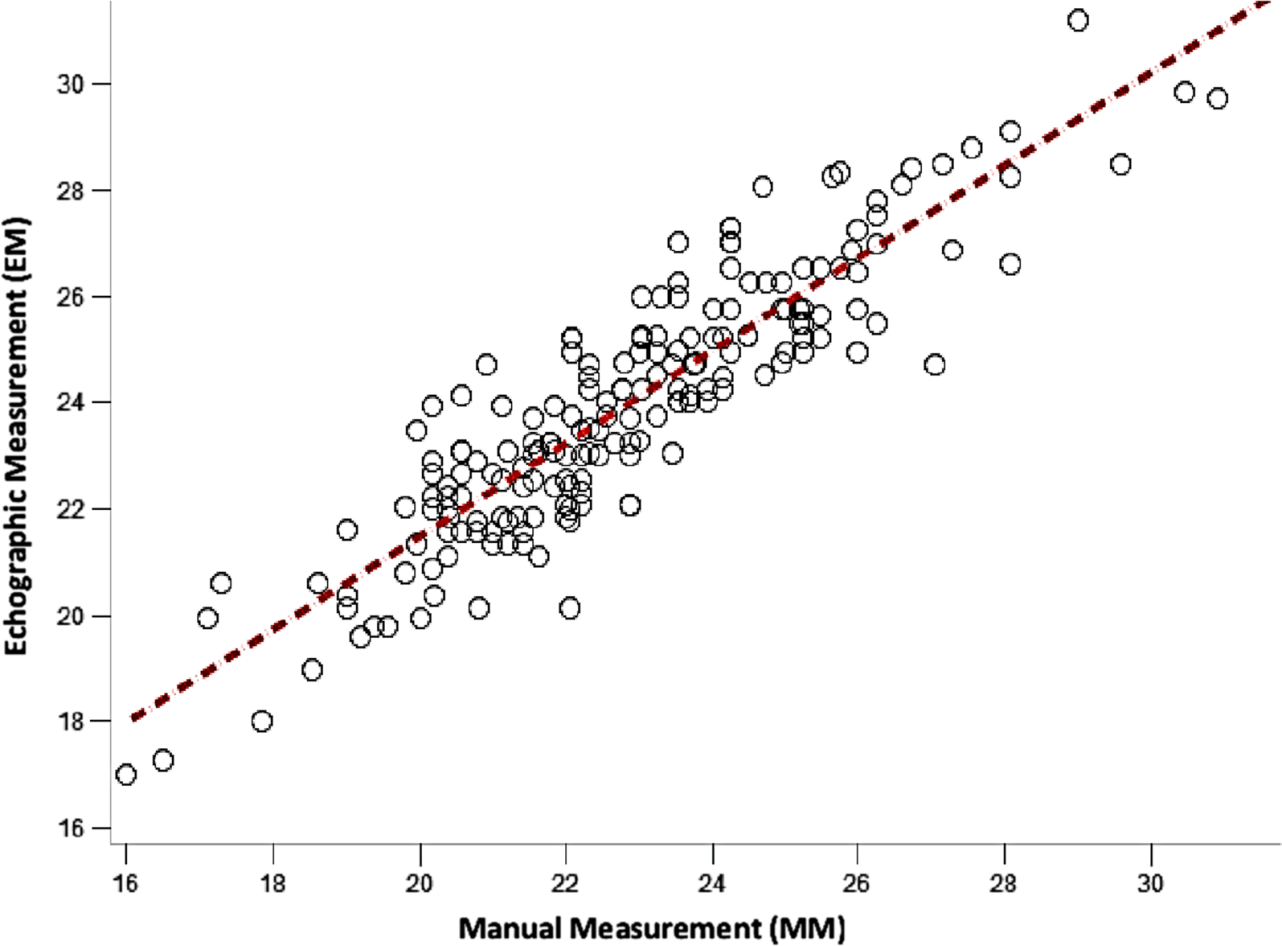
Correlation between echographic and manual measurements (MM and EM)

We therefore highlighted a strong positive relationship between echocardiographic measurements and manual measurements of the mitral cord. The coefficient of determination r^2^ is 0.805 (Fig. 5).

### Simple linear regression

We therefore proceeded to analyze a simple linear regression in order to model the relationship between our two variables (MM and EM).

The relationship between the two variables is linear (the point cloud is best summarized by a line of equation Y = aX + b). The application condition is verified, it is therefore possible to use the correlation coefficient and the simple linear regression to quantify the link between the 2 variables (EM as dependent variable and MM independent variable).

**Fig. 6:**
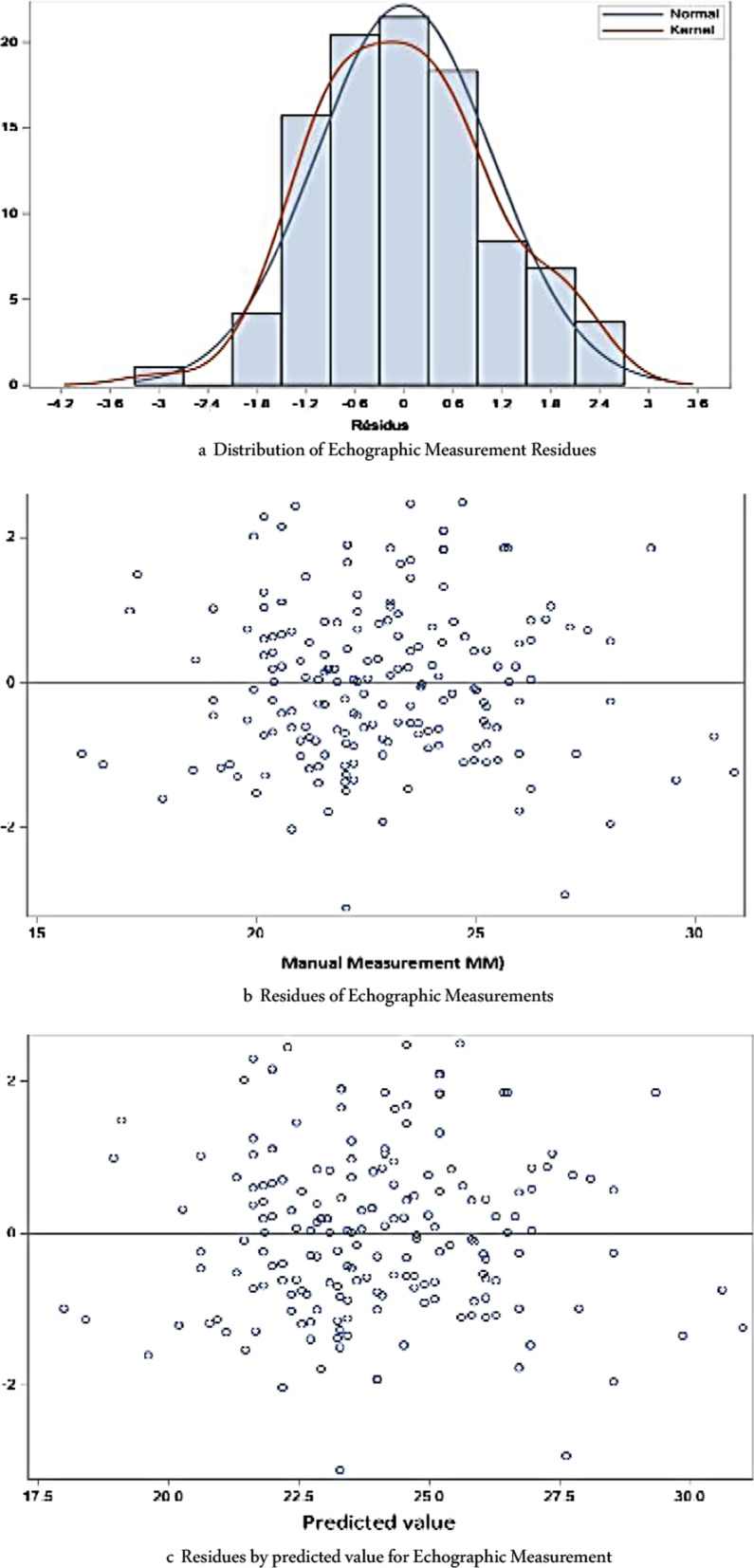
**a** Distribution of echographic measurement residues (validate the model). **b** Residues of echographic measurements (validate the model). **c** Residues by predicted value for echographic measurement (validate the model)

Graphical analysis shows that the residues visually follow a normal distribution (Fig. 6).

Errors are therefore distributed normally. The coefficient of determination R^2^ which measures the percentage of variability in ultrasound values according to manual values is 80.5%, so the model seems adequate.

**Fig. 7:**
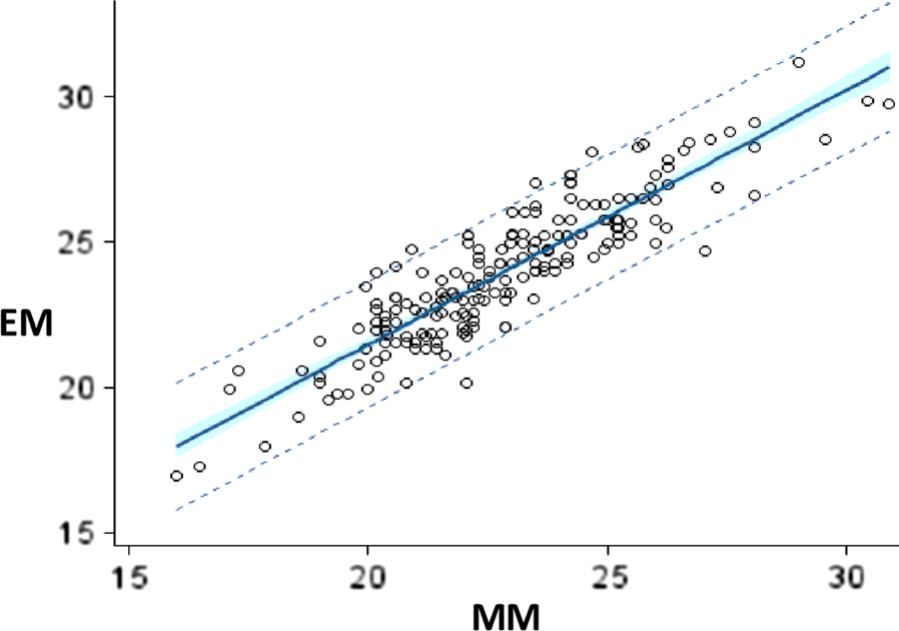
Adjustment curve for echographic measurements (ME) according to manual measurements (MM)

The adjustment curve of the dependent variable (EM) compared to the independent variable (MM) clearly shows good positive linearity (Fig. 7).

The results of the linear regression lead us to the following mathematical model:

$${\text{Y}} = {\text{aX}} + {\text{b}} \to {\text{Y}} = 0.87 \cdot {\text{X}} + 4$$

where Y is the dependent variable: echocardiographic measurement (EM) to predict; X is the independent variable: manual measurement (MM) as a predictor variable; a is the slope of the regression line and b is the intercept.

This model gave an equation by the regression line with a better fit which is as follows: EM = 0.87 ∙ MM + 4.

The EM model = 0.87 ∙ MM + 4, allows us to predict, for example, that for a manual measurement of a 20 mm chordae, the estimated echocardiographic measurement would be 21.4 mm, which is a significant correction for good surgical repair of the mitral valve.

## Discussion

The need for a reliable measurement of chordae in ventricular dynamics is necessary for a perfect anatomical repair of the mitral valve.

Esophageal echocardiographic measurement is sometimes contraindicated or impossible to perform.

These contraindications were listed previously in our introduction, however unfavorable anatomical conditions are also known [4, 5]. If 100% of the hearts studied in different studies show the consistency of an anterior papillary muscle and a posterior papillary muscle, there is, however, significant variability in the number and shape of the bellies of these papillary muscles. Usually, there are 2 papillary muscles (anterior and posterior) however each of these muscles has 2 parts: a vertex dedicated to the chordae of the large valve (anterior) and a vertex dedicated to those of the small valve (posterior). The fixation of the artificial chordae must be done at the border between the fibrous part and the muscular part of the papillary muscle. This area is just 2 mm below the top of each papillary muscle. The suturing of the artificial chordae must be performed in this area and not in the muscle itself because the fragility of the muscle would frequently lead to a risk of disinsertion.

In order to accurately predict a measurement, it is essential to use an appropriate methodology to perform adequate modeling.

Several authors, [2, 3, 6–8] presented works with different methods of study and analysis.

In this article, we have suggested criteria for the design of studies aiming to collect data to arrive at a standard model whose application makes it possible to predict the length of the echographic measurements on heart observed in dynamics from the corresponding lengths of our manual measurements on a stopped heart. We also presented an analysis strategy to produce graphs from our data. This modeling of our manual measurement is necessary. It allows us to carry out with very great precision our substitution of chordae allowing not only to effectively correct mitral leakage but also to obtain better valve clearance by preserving a harmonious bivalvular movement [1]. Manual measurement remains an anatomical measurement, however it cannot be used as such during the production of artificial chordae, it must be corrected by the proposed formula: EM: 0.87 ∙ MM + 4, in order to obtain perfect coaptation. If the 2 valves are prolapsed, we use a reference point which never prolapses and which is located at the level of the commissure (anterior or posterior). At the end of the repair, in the operating room, an echo is performed and if there is the slightest residual leak, the left atrium is reopened to correct the hiatus. No residual leak is tolerated at the end of the intervention. It is precisely to simplify the measurements and standardize the technique for calculating the length of artificial chordae that we propose this statistical analysis. In view of a very large number of valve repairs, this analysis seems relevant to us.

The MM always remain lower than the EM which are carried out under the physiological conditions of myocardial contractility.

## Conclusion

The necessary length of Gore-Tex® braids should be estimated as far as possible using transesophageal echocardiography because of the more physiological nature of the measurement and the risk of residual mitral leakage in the event of a measurement estimated manual. When patients have a formal ETO contraindication, or when ETO echocardiographic measurement is impossible, it is therefore necessary to be able to accurately predict this echocardiographic measurement from manual measurement in order to perform the substitution of chordae with very high precision.

If the TEE is often necessary to predict the feasibility of the mitral plasty, it is no longer essential in immediate preoperative to predict the size of neo-chordae necessary for repair. Indeed, the modeling of the length of the mitral valve cords by the EM = 0.87 ∙ MM + 4 model eventually makes it possible to overcome this echocardiographic measurement.

This modeling takes into account the dynamic conditions (filling of the beating heart) which are lacking during Manual Measurements during the operation.

Therefore, in case of contraindication to ETO or in case of difficult measurements in ETO, the application of the model obtained will make it possible to perform a mitral valvuloplasty with reliable results.

## Data Availability

The datasets used and analysed during the current study are available from the corresponding author on reasonable request.

## Appendix

**Table:**

d.d.l	α			
	0.10	0.05	0.02	0.01
1	0.9877	0.9969	0.9995	0.9999
2	0.9000	0.9500	0.9800	0.9900
3	0.8054	0.8783	0.9343	0.9587
4	0.7293	0.8114	0.8822	0.9172
5	0.6694	0.7545	0.8329	0.8745
6	0.6215	0.7067	0.7887	0.8343
7	0.5822	0.6664	0.7498	0.7977
8	0.5494	0.6319	0.7155	0.7646
9	0.5214	0.6021	0.6851	0.7348
10	0.4973	0.5760	0.6581	0.7079
11	0.4762	0.5529	0.6339	0.6835
12	0.4575	0.5324	0.6120	0.6614
13	0.4409	0.5139	0.5923	0.6411
14	0.4259	0.4973	0.5742	0.6226
15	0.4124	0.4821	0.5577	0.6055
16	0.4000	0.4683	0.5425	0.5897
17	0.3887	0.4555	0.5285	0.5751
18	0.3783	0.4438	0.5155	0.5614
19	0.3687	0.4329	0.5034	0.5487
20	0.3598	0.4227	0.4921	0.5368
25	0.3233	0.3809	0.4451	0.4869
30	0.2960	0.3494	0.4093	0.4487
35	0.2746	0.3246	0.3810	0.4182
40	0.2573	0.3044	0.3578	0.3932
45	0.2428	0.2875	0.3384	0.3721
50	0.2306	0.2732	0.3218	0.3541
60	0.2108	0.2500	0.2948	0.3248
70	0.1954	0.2319	0.2737	0.3017
80	0.1829	0.2172	0.2565	0.2830
90	0.1726	0.2050	0.2422	0.2673
100	0.1638	0.1946	0.2301	0.2540

Correlation coefficient table (Schwartz, Lazar and Papoz, 1995; p.125)

## Abbreviations

TEE: Trans-Esophageal Echocardiography
EM: Echographic measurement
MM: Manual measurement
ECC: Extra-Corporeal Circulation
SMM: Skewness Manual Measurement
KMM: Kurtosis Manual Measurement
Ho: Null Hypothesis
H1: Alternative Hypothesis
r: Correlation Coefficient
N: Total population
α: Alpha risk
